# Simulation of Temperature Distribution and Microstructure Evolution in the Molten Pool of GTAW Ti-6Al-4V Alloy

**DOI:** 10.3390/ma11112288

**Published:** 2018-11-15

**Authors:** Min Zhang, Yulan Zhou, Chao Huang, Qiaoling Chu, Wenhui Zhang, Jihong Li

**Affiliations:** School of Materials and Engineering, Xi’an University of Technology, Xi’an 710048, China; zhouyulan@163.com (Y.Z.); 18829028936@163.com (C.H.); 13352413558@163.com (W.Z.); chuqiaoling@xaut.edu.cn (Q.C.); lijihong@xaut.edu.cn (J.L.)

**Keywords:** temperature field, dendritic morphology, finite element, solute concentration, cellular automaton

## Abstract

In this paper, a three-dimensional (3D) finite element model was established by ABAQUS software to simulate the welding temperature field of a Ti-6Al-4V alloy under different welding currents based on a Gaussian heat source model. The model uses thermo-mechanical coupling analysis and takes into account the effects of convection and radiation on all weld surfaces. The microstructure evolution of the molten pool was calculated using the macro-micro coupling cellular automaton-finite different (CA-FD) method. It was found that with the increase of the welding current, the temperature in the central region of the moving heat source was improved and the weld bead became wider. Then, the dendritic morphology and solute concentration of the columnar to equiaxed transition (CET) in the weld molten pool was investigated. It is shown that fine equiaxed crystals formed around the columnar crystals tips during solidification. The coarse columnar crystals are produced with priority in the molten pool and their growth direction is in line with the direction of the negative temperature gradient. The effectiveness of the model was verified by gas tungsten arc welding experiments.

## 1. Introduction

Nowadays, titanium alloys, especially Ti-6Al-4V (TC4), have acquired extensive applications in aerospace, aircraft, automotive, biomedical, and chemical industries [1,2,3]. This is primarily due to their superior performance characteristics, such as low density, good corrosion resistance, high heat resistance, and high specific stiffness and specific strength. In many fields of manufacturing and processing industry, gas tungsten arc welding (GTAW) [4,5] plays a critical role, which is viewed as one of the traditional and significant material processing techniques. The mechanical and physical properties of titanium alloys are greatly affected by the microstructure evolution during molten solidification [6]. In particular, Kou [7] proposed that the dramatic transformation of temperature and solute concentration directly gave rise to an unfavorable influence on the dendritic growth. Salimi [8] developed a 3D transient analytical solution to the heat conduction problem in different plates with a circular moving heat source. The analytical results were validated by the finite element (FE) method and experiments. During metal solidification, to simulate temperature field change and microstructure transition for a laser-based additive manufacturing processing, a successful cellular automaton-finite element (CA-FE) model was proposed by Zhang [9]. Specially, it should be noted that the direct observation and measurement of the dynamic solidification process of the welding molten pool is very difficult to achieve in experiments. 

With the continuous advancement of computational technology, several approaches have been developed for modeling microstructure evolution in the weld molten pool. These approaches mainly include Monte Carlo (MC), phase field (PF), and cellular automata (CA). Anderson [10] uncovered the features of crystal growth and studied the relationship between the dendritic growth and undercooling by the MC method. Then, the temperature of the weld process was integrated with the simulation of grain growth in a computationally efficient manner [11]. However, due to lacking a physical basis, this MC method failed to perform quantitative analysis, which also greatly limited its application. Another employed a phase field (PF) method to quantitatively simulate the dendritic growth process. Qin et al. [12] simulated the solidification of a multicomponent and developed the multiphase systems based on the PF model. To simulate microstructure morphology and solute distributions of the Al-4 wt% Cu alloy in a welding molten pool under transient conditions, Wang et al. [13] developed a quantitative phase field model. However, this model needs a huge amount of computational time, resulting from requiring an extremely fine mesh. To this end, Rappaz et al. [14] proposed a CA method which characterizes the discrete temporal and spatial microstructure evolution using a network of regular cells. In the gas tungsten arc welding (GTAW) process, Zhan et al. [15,16] simulated the dendrite morphology based on the CA method. In addition, the CA method has two advantages: low computational cost and ease of coupling with the macroscopic thermal model considering the transfer of heat and mass in complex geometries. During the solidification of the TC4 alloy, Wang et al. [17] investigated the grain microstructure by means of a method where CA was coupled with FE in the molten pool. Chen et al. [18] proposed a 3D CA-FE model to permit the simulation of grain structure solidification during multiple passes of gas tungsten arc welding (GTAW) and gas metal arc welding (GMAW). Here, the CA-FE method was better used for simulating the temperature distribution and microstructure formulation in the molten pool. To date, there has been little investigation on predicting the temperature distribution and dendritic morphology of the columnar to equiaxed transition (CET) and obtaining experimental validation during Ti-6Al-4V alloy gas tungsten arc welding.

In this study, the temperature field and dendritic morphology were simulated by a CA-FD method. The FE software ABAQUS (version 6.13, Dassault SIMULIA, Providence, RI, USA) was used to compute the thermal field evolution under different welding current conditions. Moreover, the CA model was built to simulate the microstructure evolution of the CET process. The simulated results were verified by corresponding experiments.

## 2. Thermal Modeling of Welding

**Figure 1:**
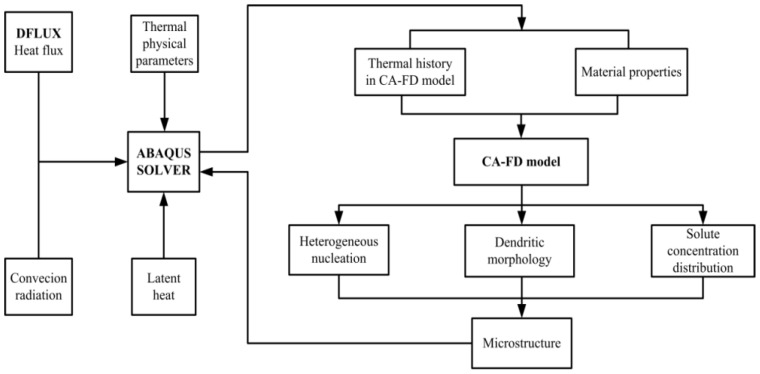
The flow chart of the coupled CA-FD model.

A visual thermal modeling of the welding process was performed to predict the thermal fields in the solidification area, by using the FE software ABAQUS and its user subroutines. The subroutines were implemented by the FORTRAN programmable language and linked to the ABAQUS software. The entire procedure of the coupling analysis is shown in Figure 1.

**Figure 2:**
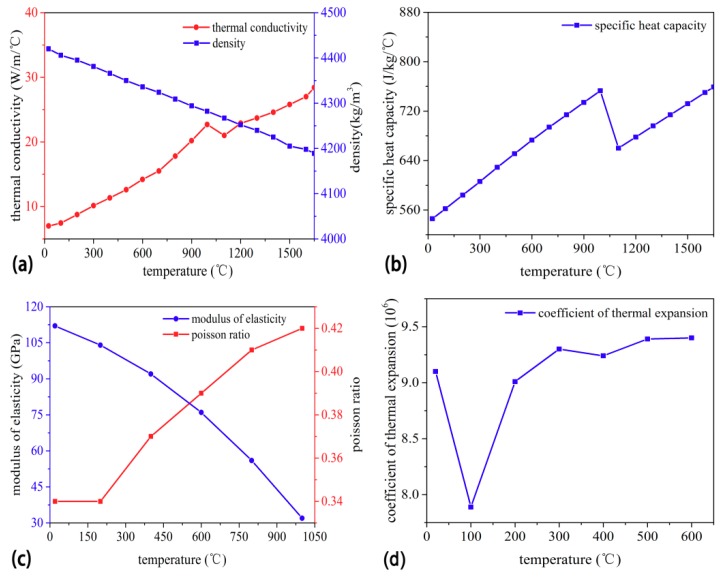
The material properties used to simulate temperature distribution: (**a**) thermal conductivity, (**b**) specific heat capacity, (**c**) elasticity modulus and Poisson ratio, and (**d**) thermal expansion coefficient.

A TC4 alloy plate was used; its chemical compositions are listed in Table 1 and the material properties [19] used for thermal numerical simulation are shown in Figure 2.

### 2.1. Meshing and Analysis Settings

**Figure 3:**
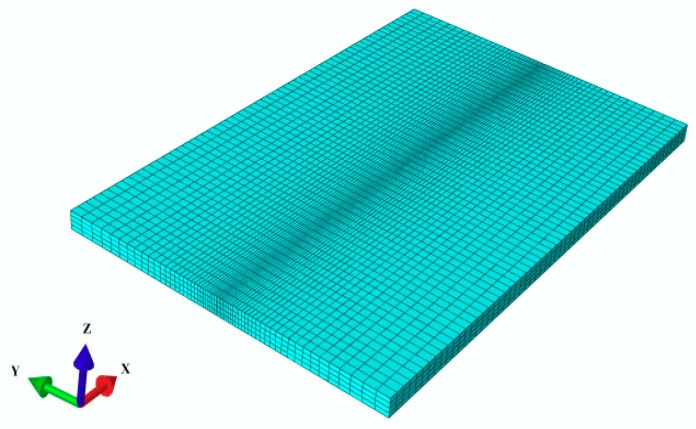
The FE model used in the numerical analysis.

The TC4 plate (120 mm × 80 mm × 4 mm) was melted in the centerline by using a gas tungsten arc welding (GTAW) process without filler metal. The dimensions of the simulated welded thin plate were exactly identical to those used in the welding experiments. A total of 14,400 C3D8T elements were used in the simulation. In C3D8T, C represents a solid element, 3D indicates three-dimensional, and 8T denotes eight nodes. The FE model used is shown in Figure 3.

It can be easily seen that the mesh in the central area of the weld bead is relatively fine and the other areas are sparse to obtain higher computation accuracy. In addition, the analysis type uses thermo-mechanical coupling analysis with two analysis steps: a heating analysis step and a cooling analysis step.

### 2.2. Heat Transfer Equation

The welded sample is viewed as a solid subjected to conduction heat transfer, with boundary conditions to model heat transfer between the sample and the surrounding environment. Then, the three-dimensional transient thermal equation is given as follows [20]:

(1) $$\rho C_{p}\frac{\partial T}{\partial t}=\frac{\partial }{\partial x}(k\frac{\partial T}{\partial x})+\frac{\partial }{\partial y}(k\frac{\partial T}{\partial y})+\frac{\partial }{\partial z}(k\frac{\partial T}{\partial z})+Q_{v}$$

where T is temperature, ρ is material density, $C_{P}$ is specific heat, k is thermal conductivity, and $Q_{v}$ represents volumetric heat flux in $W/m^{3}$. To solve the heat equation, the thermal conductivity, density, and specific heat are required. 

### 2.3. Heat Source

**Figure 4:**
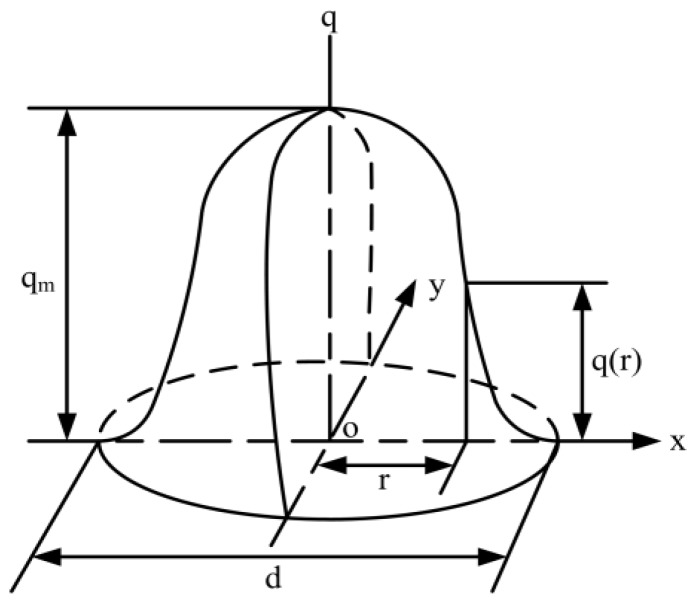
The Gaussian heat source model.

The heat source was exerted by a heat flow density imposed on the sample surface interacting with the arc. A Gaussian heat source model was chosen (shown in Figure 4), centered on the arc axis, moving in translation along the x-axis at the welding speed $v_{s}$. It should be noted that o represents the coordinate center of the xy plane.

The heat flow density distribution in the surface is then given by [20]:

(2) $$q(r)=\frac{2q_{m}}{\pi d}\exp\frac{-3r^{2}}{d^{2}}\;$$

where $q_{m}$ is Gaussian amplitude, d is width, and *r* represents the distance to the center of the heat source, defined by:

(3) $$r={\left(X-X_{0}-v_{s}t\right)}^{2}+{\left(Y-Y_{0}\right)}^{2}\;$$

Here, $X_{0}$ and $Y_{0}$ are the coordinates of the initial position of the heat source and t denotes the time.

The total power transmitted to the sample by this distribution, obtained by integrating Equation (2) for r from 0 to infinity, must match the effective welding power. The relationship between the distribution parameters and process parameters can be given as

(4) $$q_{m}=\eta \cdot UI\;$$

where *U* is the welding voltage, *I* is the welding current, and η indicates the welding efficiency.

### 2.4. Boundary and Initial Conditions

At the beginning, the initial and ambient temperatures of the FE model for all simulations were set to 25 °C. The thermal boundary conditions mainly include convection in air, radiation from the surface of the workpiece toward air in light of the Stefan-Boltzmann relationship, and conduction from the workpiece toward the metal support. The heat loss from surface convection and radiation can be given as [20]:

(5) $$q_{\mathrm{conv}}=h\left(T_{c}-T_{0}\right)\;$$

(6) $$q_{\mathrm{rad}}=\varepsilon \sigma \left[{\left(T_{c}-T_{\mathrm{abs}}\right)}^{4}-{\left(T_{0}-T_{\mathrm{abs}}\right)}^{4}\right]\;$$

Here, $T_{c}$ is the current temperature, $T_{0}$ indicates the ambient temperature, and $T_{\mathrm{abs}}$ is the absolute zero temperature. In addition, ε represents the emissivity and σ is the Stefan-Boltzmann constant, which has the value of $5.68\;\times \;10^{-8}\;J/K^{4}m^{2}s$. In this paper, the convection coefficient is taken as 8 and the emissivity is 0.85, and the heat transfer coefficient has the value of $h=10\;W/m^{2}\cdot K$.

### 2.5. Macro–Micro Coupling of the Temperature Field

**Figure 5:**
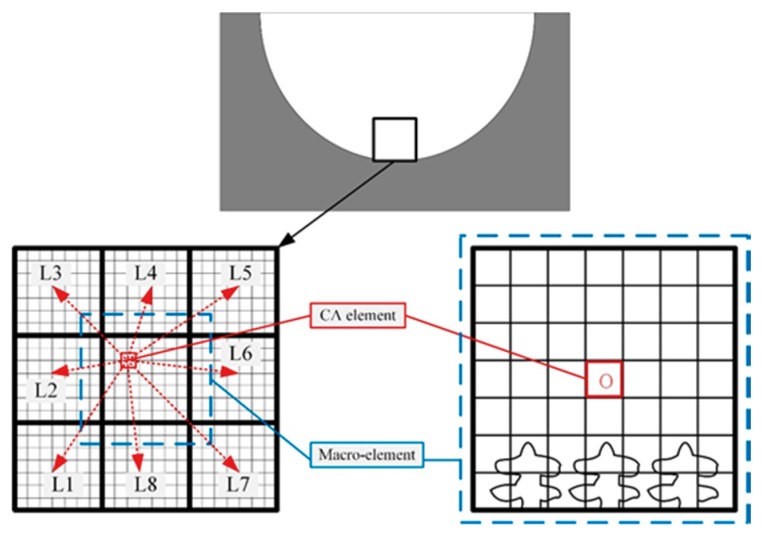
The macroscopic and microscopic coupling analysis.

The calculation of the welding heat transfer process is the basis of the microstructure simulation. However, the calculation of the heat transfer process of the weld pool is performed on a macro scale, while the calculation of the dendrite growth based on the CA method is carried out on a micro scale. Therefore, it is necessary to establish a macro-micro coupling model for temperature field calculation. The macroscopic temperature field was solved by the finite difference (FD) method using the ABAQUS finite element software. The CA model was built to simulate the microstructure evolution of the columnar to equiaxed transition (CET) process. The temperature of the CA element is affected by the temperature of the macro elements around it. It is related to the distance from the central node of the element to the surrounding macro elements as shown in Figure 5.

The temperature value of the CA element can be expressed by the following formula [16]:

(7) $$To=\sum_{i=1}^{N}Li^{-1}Ti/\sum_{i=1}^{N}Li^{-1}\;$$

where *T*o is the temperature of the micro-element O; *Ti* is the macro-element temperature around the point O; *Li* is the distance from the point O to the surrounding macro-element; and *N* is the number of macro-elements around the micro-element, the value of which is 8.

## 3. Modeling of the Dendritic Growth

The growth of columnar grains and equiaxed grains was simulated in the modeling. The CET can be calculated when the volume fraction for equiaxed grains reached a given limit at the solidification front. The thermal properties of the material used in the present simulation are shown in Table 2 [18].

### 3.1. Dendritic Nucleus Model

During the solidification process of the molten pool, the interface between the molten pool of liquid and the solid substrate is the nucleation surface affiliated with the grains’ growth in the molten pool. A heterogeneous nucleation method was used to simulate the solidification evolution process of the grains in the molten pool. A quasi-continuous nucleation model was established based on the Gaussian distribution function. The term *dn/d*(∆*T*) was used to describe the variation of the grains’ nucleation density, and the total density of nuclei n(ΔT) formed at a certain undercooling ΔT was given as [21]:

(8) $$n(\Delta T)=\int_{0}^{\Delta T}\frac{dn}{d\left(\Delta T\right)}d\left(\Delta T\right)\;$$

The change rate of nucleation density varies with the supercooling degree satisfied with Gaussian distribution. It can be calculated by the following expression:

(9) $$\frac{dn}{d\left(\Delta T\right)}=\frac{n_{\max}}{\sqrt{2\pi }\Delta T}\exp\left[-\frac{1}{2}{\left(\frac{\Delta T-\Delta T_{N}}{\Delta T_{\sigma }}\right)}^{2}\right]\;$$

where $n_{\max}$ is the maximum nucleation density and $\Delta T_{\sigma }$ is the standard deviation of undercooling. In the calculation process, the nucleation point location was randomly chosen.

### 3.2. Solute Diffusion Model

For binary alloys or multicomponent alloys, the solute diffusion also plays an important role on dendrite growth in the weld molten pool and the solute concentration gradient is the driving force for solute diffusion in the solid and liquid phases. Then, the solute concentration in the solid and liquid phases is determined by solving the governing equation for each phase separately, as follows:

(10) $$\frac{\partial C_{i}}{\partial t}=D_{i}\nabla^{2}C_{i}+C_{i}\cdot (1-k)\frac{\partial f_{s}}{\partial t}\;$$

where C is the solute concentration, D represents the solute diffusivity, the subscript i denotes a solid or liquid, and k is the partition coefficient.

At the solid–liquid interface, the solute partition between the liquid and solid is given by:

(11) $${C_{s}}^{*}=k\cdot {C_{l}}^{*}\;$$

where ${C_{s}}^{*}$ and $C_{l}^{*}$ denote the interface solute concentrations in the solid and liquid phases, respectively.

Based on the counting method advanced by Nastac [22], the interface curvature of a cell with a solid fraction can be derived by calculating the nearest and second-nearest neighboring cells:

(12) $$K=\frac{1}{l_{\mathrm{CA}}}(1-2\frac{f_{s}+\sum_{j=1}^{N}f_{s}(j)}{N+1})\;$$

where $l_{\mathrm{CA}}$ represents the length of the CA cell side, N is the number of the nearest and the second-nearest neighboring cells, and $f_{s}(j)$ is the solid fraction of neighboring cells.

The liquid concentration in the interface cell is given as [23]:

(13) $$C_{l}={C_{l}}^{*}-\frac{1-f_{s}}{2}l_{\mathrm{CA}}\cdot G_{c}\;$$

where $G_{c}$ is the concentration gradient in front of the solid-liquid interface, and the interface equilibrium composition ${C_{l}}^{*}$ is obtained by:

(14) $${C_{l}}^{*}=C_{0}+\frac{1}{m_{l}}\left[T^{*}-{T^{eq}}_{l}+\Gamma K\right]\;$$

where $C_{0}$ indicates the initial solute concentration, $T^{*}$ is the interface equilibrium temperature calculated by Equation (1), $T_{1}^{\mathrm{eq}}$ is the equilibrium liquidus temperature at $C_{0}$, $m_{l}$ is the liquidus slope, Γ is the Gibbs-Thomson coefficient, and K is the average curvature of the liquid-solid interface.

### 3.3. Undercooling

Kurz et al. [24] developed the KGT (Kurz-Giovanola-Trivedi) model. It was introduced to simulate and calculate the growth process of the dendrite tip. De-Chang et al. [25] proposed that the undercooling of the solid–liquid interface mainly includes temperature, concentration, and curvature in the CA model. The anisotropy of the interface energy has a great effect on the curvature undercooling, so the model must take the interface anisotropy into consideration. At the same time, the degree of undercooling in the solid–liquid interface prerequisite is calculated as [26]:

(15) $$\Delta T(t_{n})=T^{\prime }-T_{i,j}+m_{l}(C_{0}-{C_{l}}^{*})+\Gamma K(t_{n})\{1-15G_{x}\cos[4(\theta -\theta_{0})]\}\;$$

where $T^{\prime }$ is the temperature at the interface, $T_{i,j}$ is the temperature of the undercooled melt, $C_{0}$ is the initial solute concentration, ${C_{l}}^{*}$ is the solute concentration of the liquid at the interface, and $m_{l}$ is the slope of the liquidus. Besides, Γ is the Gibbs–Thompson coefficient and $K(t_{n})$ is the interface curvature calculated from Equation (12). $G_{x}$ is the anisotropy intensity of the liquid–solid interface.

(16) $$\theta_{0}=\cos^{-1}\left(\frac{\partial f_{s}/\partial x}{{\left({\left(\partial f_{s}/\partial x\right)}^{2}+{\left(\partial f_{s}/\partial y\right)}^{2}\right)}^{1/2}}\right)\;$$

(17) $$\theta =\arctan(\frac{\partial f_{s}/\partial y}{\partial f_{s}/\partial x})\;$$

Then, $\theta_{0}$ represents the angle between the growth direction of the dendrites and the positive direction of the coordinate axis, and it can be calculated from Equation (16). Likewise, θ is also the angle between the normal of the solid–liquid interface and the positive direction of the coordinate axis, which is derived from Equation (17). As the heat undercooling is relatively small, the effect of dynamics on high dendritic growth is not taken into account and the solute diffusion in the solid phase can be neglected.

### 3.4. Selection of Time Step

In order to ensure stability in the calculation of the composition field and the advance rate of the solid–liquid interface, the time step is determined by the following Equation [27]:

(18) $$\Delta t\leq \frac{1}{5}\min(\frac{\Delta x^{2}}{D_{l}},\frac{\Delta x}{V_{\max}})\;$$

where Δx is the grid size, $D_{l}$ is the solute diffusion coefficient in the liquid, and $V_{\max}$ represents the welding speed.

### 3.5. Experimental Details

For the purpose of validating the feasibility and accuracy of the presented model, the welding experiment was automatically carried out by an YC-400TX GTAW machine (Panasonic Welding Systems (Tangshan) Co., Ltd, Tangshan, China). The welding parameters used are shown in Table 3. It should be pointed that the welding efficiency in Table 3 was set as 0.8 [28]. Then, the specimens were mechanically ground with 120-grit SiC paper, were etched in a solution composed of 400 mL H_2_O + 40 g KOH + 40 mL H_2_O_2_, and purged with an ultrasonic cleaner in order to remove surface contaminants. 

An accurate simulation for the moving heat source of the weld process is a prerequisite to correctly predict the temperature distribution and dendritic growth. The parameters’ calibration for the heat source, including the peak temperature, shape, and dimensions of the welding molten pool, is performed by optimizing parameters such as the welding voltage and the welding current. The parameter optimization is based on satisfying the macrograph experimentally observed for a welding cross section. 

**Figure 6:**
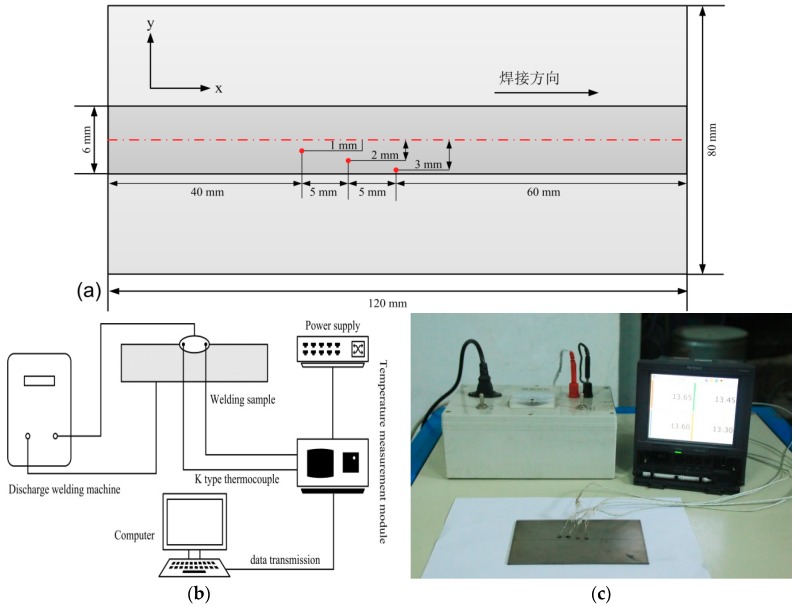
Testing of the welding thermal cycle: (**a**) the welding sample geometry, (**b**) the diagram of the test platform, and (**c**) the picture of the test platform.

The calculated temperature field was validated by means of the K-type thermocouple measurement of the transient temperature captured at corresponding test points during the actual welding process, as shown in Figure 6a. In order to evaluate the accuracy for the simulated temperature distribution results, the test platform of the welding thermal cycle was built as shown in Figure 6b. It primarily consisted of a stored energy welding machine (self-developed), welding sample, K-type thermocouple, temperature measurement module (TR-W500, KEYENCE Corporation, Osaka, Japan), and computer. Since it is very hard to put the thermocouple very close to the welding sample surface without surpassing the temperature limitation for the thermocouple, the temperature distribution was measured at a smaller distance from the heat affected zone (HAZ). Hence, the K-type sheathed thermocouples were directly embedded in the welding sample surface by using the stored energy welding machine. The embedded thermocouples were located at four points on the workpiece surface; as shown in Figure 6c, these four points were evenly spaced along the welding direction.

## 4. Results and Discussions

### 4.1. Temperature Distribution and Weld Bead Geometry

**Figure 7:**
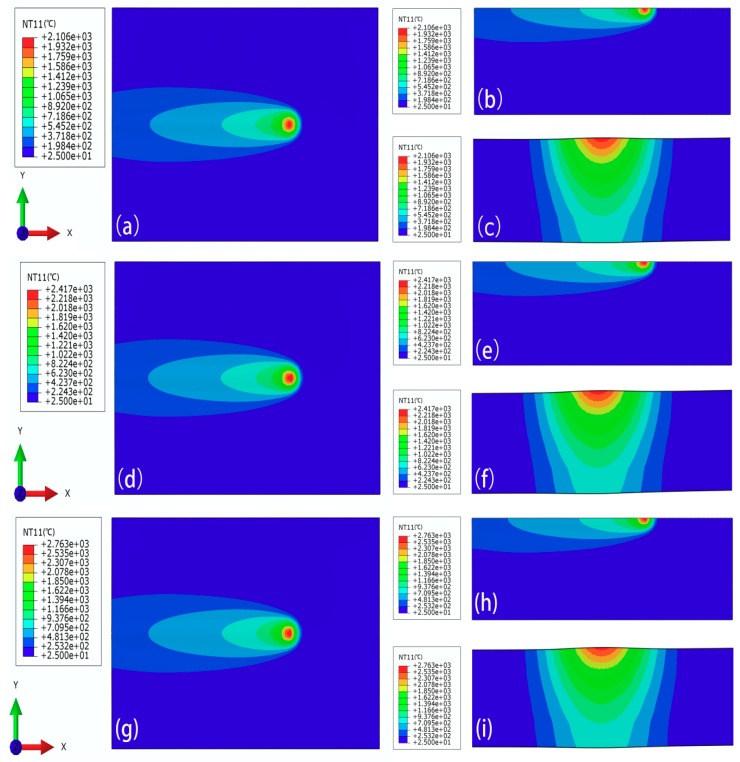
The temperature fields under different welding currents at t = 16 s, with (**a**) the xy plane at I = 60 A, (**b**) the half xy plane at I = 60 A, (**c**) the yz plane at I = 60 A; (**d**) the xy plane at I = 75 A, (**e**) the half xy plane at I = 75, (**f**) the yz plane at I = 75 A, (**g**) the xy plane at I = 90 A, (**h**) the half xy plane at I = 90 A, and (**i**) the yz plane at I = 90 A.

The temperature distributions in the welding process were calculated under different welding currents, including 60 A, 75 A, and 90 A. The specific temperature distribution results are shown in Figure 7. It should be noted that the welding voltage is 13.8 V, the welding speed is 5 mm/s, and the welding efficiency is 0.8. According to Equation (4), the welding input power can be derived.

**Figure 8:**
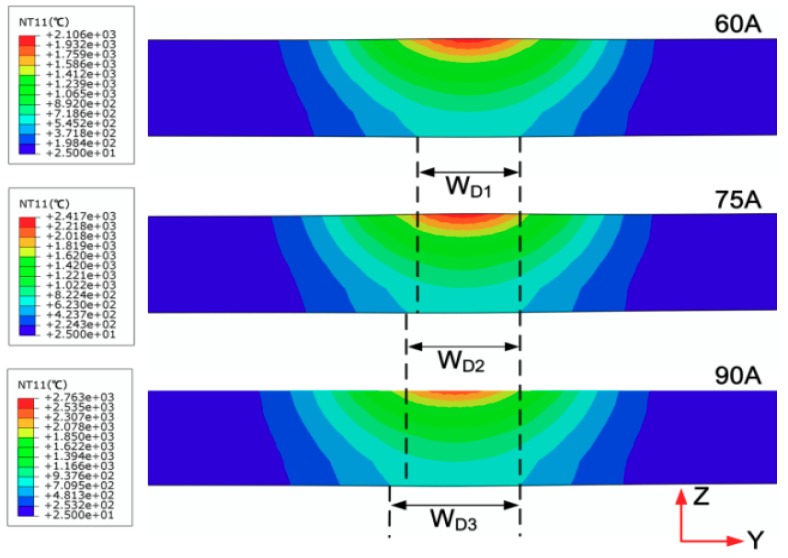
The cross sections of the welding bead under different welding currents.

From Figure 7, it can be seen that as the welding heat source moves, the molten pool advances stably and the shape of the molten pool remains substantially unchanged. When the welding current is gradually increased, the temperature in the central region of the moving heat source is increased. To further investigate the relationship between the welding current and the welding bead geometry, the cross sections of the welding bead under different welding currents were observed, as shown in Figure 8. Then the bottom width of weld bead is represented by W_D_. 

From Figure 8, it can be found that as the welding current increases, the temperature of the central region of the moving heat source increases and the weld transverse cross section becomes wider, i.e., $W_{D_{l}}>W_{D_{2}}>W_{D_{3}}$. In addition, the temperature in the central region of the moving heat source is larger than the melting point of the titanium alloy (1650 °C).

**Figure 9:**
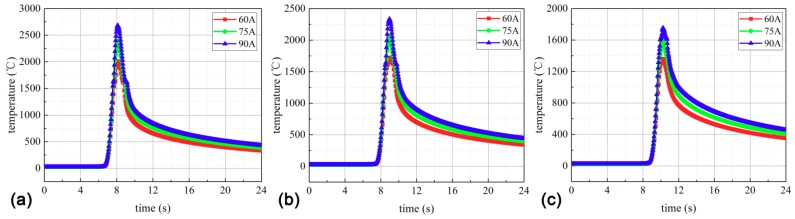
The simulated thermal cycle curves at different distances from the weld center: (**a**) 1 mm, (**b**) 2 mm, and (**c**) 3 mm.

In order to further analyze the effect of the welding current on the temperature distribution during the welding, the thermal cycle curves were obtained, as shown in Figure 9. From Figure 9, it can be found that due to the rapid heating rate of the welding, the curve rises extremely fast and the temperature rapidly reaches a peak; the closer the weld bead is, the faster the temperature rises and the higher the peak temperature. During the cooling phase, the temperature drops relatively slowly. In addition, the simulated temperature values under different distances and currents are listed in Table 4.

From Table 4, it can be found that the temperature value is lower than the melting point of the titanium alloy (1650 °C) under L = 3 mm and I = 60 A. However, the temperature is larger than the melting point of the titanium alloy under L = 3 mm and I = 90 A. Then, the welding heat input *P* can be calculated thus:

(19) $$P=\frac{\eta UI}{V}\;$$

Here, *U* is the welding voltage, *I* is the welding current, *V* represents the welding speed, and η is the welding efficiency. According to Equation (19), the welding current is positively correlated with the welding heat input when *U* and *V* are determined. It should be noted that the welding efficiency is 0.8 and the welding voltage is 13.8 V in Equation (19). Then, the welding heat inputs under I = 60 A, I = 75 A, and I = 90 A are 132.48 J·mm^−1^, 165.60 J·mm^−1^, and 198.72 J·mm^−1^, respectively. When the welding heat input is too small, welding defects are easily caused; but when the welding heat input is too large, coarse columnar crystals are generated in the weld bead. This leads to the increase of the brittleness of the welded joints. Therefore, within the range of reasonable welding heat input, the weld grain size is more uniform. Based on the analysis mentioned above, the welding thermal cycle test is performed under the welding current I = 75 A.

**Figure 10:**
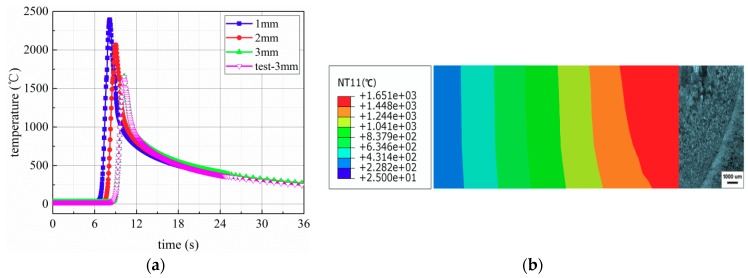
The comparison between the simulated results under I = 75 A and the experimental observations: for (**a**) thermal cycle curves and (**b**) weld bead geometry.

The calibration between the simulated results and the experimental observations is presented as shown in Figure 10. The comparative analysis of both the simulated results and experimental observations was performed. Figure 10a shows that the experimental and simulated time–temperature curves match well, including the heating rate, peak temperature, and cooling rate. Here, the heating and cooling rates are calculated by dividing the difference between the initial and current temperature values by the fixed time step. The comparison between the simulated weld bead geometry and the measured macrograph of a polished and chemically etched weld bead transverse cross section is shown in Figure 10b.

According to the temperature contour above the melting point of 1650 °C, the fusion zone is determined in the FE model. From Figure 10b, it can be found that the simulated fusion boundary isotherm is in good accordance with the experimental fusion boundary and penetration depth. It is preliminarily concluded that the weld bead transverse cross section is better when the welding current is 75 A.

### 4.2. Calculations and Measurements of the CET

**Figure 11:**
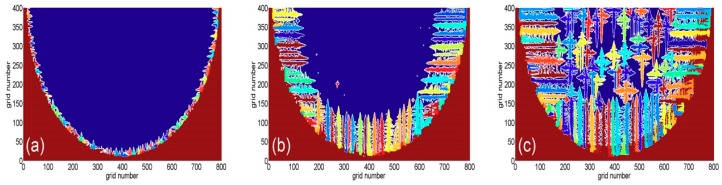
The morphology of grains in the weld pool at different times: (**a**) t = 0.065 s, (**b**) t = 0.186 s, and (**c**) t = 0.227 s.

For further evaluating the validity and rationality of the obtained welding process parameters from the welding temperature field analysis, these parameters act as the heat input parameters of the CA-FD coupling model. Then, the microstructure evolution is calculated by the CA-FD coupling model. The welding voltage is 13.8 V and the welding current is 75 A in this calculation, and the calculation areas are divided into rectangular macroscopic grids. In addition, these macroscopic grids consist of uniform microscopic grids, the grid size is 5 μm, and the time step is 0.5 ms. Of importance, the iteration number is set as 130, 372, and 454, respectively. The grains’ growth tends to be dramatically changed with the influence of heat dissipation direction. The growth morphology of the dendrites in the weld pool varies with time during the solidification, as shown in Figure 11.

From Figure 11a, it can be seen that the columnar crystals are formed at the edge of the weld pool at the beginning of solidification. With the progress of solidification, these columnar crystals continuously grow and some fine equiaxed crystals form at their tips, as shown in Figure 11b. The solidification layer continues to move inward and the solid-phase cooling capability is gradually weakened. It should be pointed that when the constitutional supercooling is high enough, the nonuniform nucleation is activated and the equiaxed crystals’ nucleation starts in the center of the molten pool. From Figure 11c, it is shown that the growth of the columnar crystals is greatly affected by the growth of the central equiaxed grains. This leads to the arrest of the longitudinal growth for the partial columnar crystals, whereas their radial growth and secondary dendrite arms are intensified. The transformation from the columnar crystals to the equiaxed crystals is eventually achieved. When the grain growth direction is perpendicular to the heat dissipation direction of the molten pool wall, columnar crystals are formed. If the heat dissipation is along all directions around the melt, equiaxed crystals are formed. It can be concluded that the morphology of the dendrites exhibits randomness and asymmetry under the effect of the altering temperature field in the molten pool.

**Figure 12:**
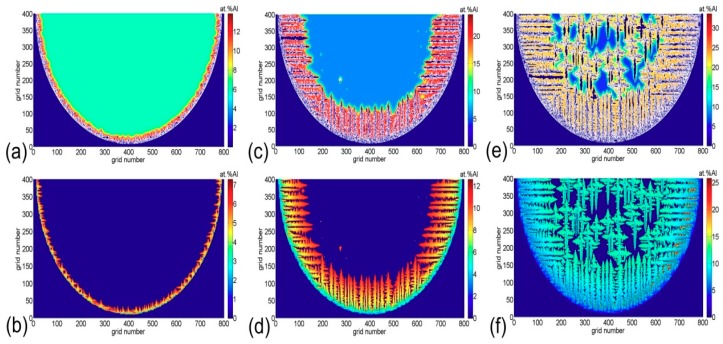
The solute distribution for solid and liquid phases in the weld pool at different times: (**a**) liquid phase at t = 0.065 s, (**b**) solid phase at t = 0.065 s, (**c**) liquid phase at t = 0.186 s, (**d**) solid phase at t = 0.186 s, (**e**) liquid phase at t = 0.227 s, and (**f**) solid phase at t = 0.227 s.

The solute concentration is the key factor of the liquid–solid state during the dendritic nucleation and growth. When the solute field is changed, the direction and morphology of the grain growth will be changed. Hence, the corresponding liquid solute concentration and solid solute concentration, as shown in Figure 12, are discussed. From Figure 12a,c,e, it can be seen that the release of solutes in the solid phase makes the liquid phase solute concentration located at the front of solid–liquid interface increase. Simultaneously, the solute diffusion rate in the liquid phase of the dendritic tip is much smaller than the dendrites’ growth speed at the solid–liquid interface. In addition, when the competitive growth of columnar and equiaxed crystals occurs, the solute fields formed by the growth of equiaxed grains and columnar crystals will affect each other.

In light of Figure 12b,d,f, it can be seen that the growth of dendrites is always followed by the segregation phenomenon when observing the distribution of solute concentration in the solid phase during the CET process. At the beginning of the CET, the region of highest solute concentration is the columnar crystal tip. When the CET transformation is conducted at the final stage, the equiaxed crystals grow well and the solid solute concentration reaches 15%. At this point, it is prone to forming regional segregation after solidification. Therefore, the evolution of columnar–equiaxed crystals is not only influenced by the growth of columnar crystals being hindered by the equiaxed grains, but also the interaction between the solid and liquid solute concentrations. 

**Figure 13:**
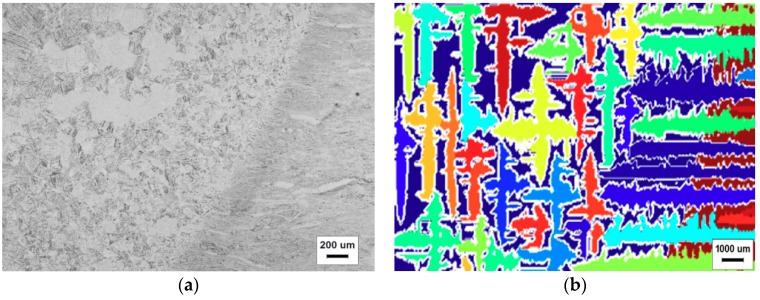
The micrograph of the TC4 alloy in the weld pool: (**a**) the test result and (**b**) the simulated result.

The cross section of the TC4 alloy welded joint is selected for the purpose of testing the validity of the simulated microstructure evolution results. It was vertical to the direction of the weld moving heat source and was ground and polished. The welding samples were etched by 10 mL HF + 20 mL HNO_3_ + 50 mL H_2_O. Microstructure observation was conducted using an inverted GX671 metallographic microscope produced by OLYMPUS (Tokyo, Japan). Owing to the whole welding pool having good symmetry, the microscopic morphology of only half of the weld pool is shown in Figure 13a.

From Figure 13a, it can be seen that the equiaxed crystals mainly concentrate in the central region of the weld pool and the CET appears in the region near the fusion line. It can be concluded that the simulated results are in good agreement with the metallographic tests.

## 5. Conclusions

In this study, based on the CA-FD model coupled with the FE model, the temperature distribution and dendritic morphology of the molten pool for TC4 alloy plates welded by GTAW were analyzed theoretically and experimentally. In addition, the effect of the welding current on weld bead geometry was analyzed in detail. In summary, the main conclusions inferred from the above analysis are as follows:

(1) In order to ensure the accuracy of the simulated temperature distribution, the developed FE model took nonlinear thermal analysis, the temperature dependency of the thermal materials’ properties, and a moving heat source into consideration. Furthermore, the convection and radiation conditions were also considered in this model.
(2) During the GTAW process, the temperature distribution in a macro region around the molten pool was calculated by the developed FE model under different welding currents. It was found that the transverse cross section of the weld bead was better when the welding current was 75 A. The obtained current parameter acted as the input parameter of the CA-FD coupling model.
(3) Then, the effect of several process conditions on the solidification microstructure was investigated by the CA-FD model, especially solidification time and temperature. It is shown that the coarse columnar crystals are produced with priority in the molten pool and their growth direction is in line with the direction of the negative temperature gradient. With the increase of temperature and solute concentration at the front of the solid-liquid interface, the columnar crystal grains show the trend of transforming to equiaxed crystals.
(4) This work contributes to the understanding of microstructure evolution and temperature characteristics in the molten pool. It can provide a fundamental basis for the selection of welding process parameters for GTAW processing of the TC4 alloy. The present model will be further enhanced to include the effect of fluid flow on dendrite growth in the molten pool.

## Figures and Tables

**Table 1:** Chemical composition of the Ti-6Al-4V (TC4) titanium alloy plate.

Element	Ti	Al	V	Fe	O
wt%	Balance	5.5–6.76	3.5–4.5	0.25	0.2

**Table 2:** Material properties’ parameters used in the microstructure simulation.

Property	Variable	Value
Liquidus temperature	$T_{L}$	1703 °C
Solidus temperature	$T_{S}$	1678 °C
Partition coefficient	$k_{0}$	0.95
Diffusion coefficient in liquid	$D_{L}$	5 × 10^−9^ m^2^/s
Diffusion coefficient in solid	$D_{S}$	5 × 10^−13^ m^2^/s
Liquidus slope	$m_{L}$	−1.4
Maximum nucleation density	$n_{\max}$	4 × 10^9^/m^3^
Standard deviation of undercooling	$\Delta T_{\sigma }$	0.5 °C
Maximum undercooling	$\Delta T_{\max}$	2 °C
Gibbs–Thomson coefficient	Γ	$3.66\;\times \;10^{-7}\;m\cdot K$
Initial concentration	$C_{0}$	6 at.%

**Table 3:** Welding procedure parameters of Ti–6Al–4V alloy plate gas tungsten arc welding (GTAW).

Parameters	Welding Speed	Welding Voltage	Welding Current	Welding Efficiency
Value	5 mm/s	13.8 V	75 A	0.8

**Table 4:** The simulated temperature maximum values under different distances and currents.

Parameters	Different Currents		
**Different Distances**	I = 60 A	I = 75 A	I = 90 A
L = 1 mm	2013.27 °C	2394.68 °C	2681.89 °C
L = 2 mm	1723.94 °C	2063.25 °C	2337.25 °C
L = 3 mm	1420.60 °C	1653.74 °C	1780.63 °C
